# Evaluating R&D efficiency of China’s listed lithium battery enterprises

**DOI:** 10.1007/s42524-022-0213-5

**Published:** 2022-08-09

**Authors:** Shizhen Bai, Xinrui Bi, Chunjia Han, Qijun Zhou, Wen-Long Shang, Mu Yang, Lin Wang, Petros Ieromonachou, Hao He

**Affiliations:** 1 School of Management, Harbin University of Commerce, Harbin, 150028 China; 2 Department of Management, Birkbeck, University of London, London, WC1E 7HX UK; 3 Department of Systems Management and Strategy, University of Greenwich, London, SE10 9LS UK; 4 Beijing Key Laboratory of Traffic Engineering, College of Metropolitan Transportation, Beijing University of Technology, Beijing, 100124 China; 5 School of Traffic and Transportation, Beijing Jiaotong University, Beijing, 100008 China; 6 Centre for Transport Studies, Imperial College London, London, SW7 2AZ UK; 7 School of Business Administration, Chongqing Technology and Business University, Chongqing, 400067 China

**Keywords:** Data Envelopment Analysis, R&D investment efficiency, China’s listed lithium battery enterprises, technical efficiency, pure technical efficiency, scale efficiency

## Abstract

Promoting the growth of the lithium battery sector has been a critical aspect of China’s energy policy in terms of achieving carbon neutrality. However, despite significant support on research and development (R&D) investments that have resulted in increasing size, the sector seems to be falling behind in technological areas. To guide future policies and understand proper ways of promoting R&D efficiency, we looked into the lithium battery industry of China. Specifically, data envelopment analysis (DEA) was used as the primary approach based on evidence from 22 listed lithium battery enterprises. The performance of the five leading players was compared with that of the industry as a whole. Results revealed little indication of a meaningful improvement in R&D efficiency throughout our sample from 2010 to 2019. However, during this period, a significant increase in R&D expenditure was witnessed. This finding was supported, as the results showed that the average technical efficiency of the 22 enterprises was 0.442, whereas the average pure technical efficiency was at 0.503, thus suggesting that they were suffering from decreasing returns to scale (DRS). In contrast, the performance of the five leading players seemed superior because their average efficiency scores were higher than the industry’s average. Moreover, they were experiencing increasing scale efficiency (IRS). We draw on these findings to suggest to policymakers that supporting technologically intensive sectors should be more than simply increasing investment scale; rather, it should also encompass assisting businesses in developing efficient managerial processes for R&D.

This work was supported by R&D and Application Demonstration of Common Key Technologies in Modern Service Industry, Key Special Sub Topics of National Key R&D Plan (Grant No. 2018YFB1402500).

### 1 Introduction

The development of electric vehicles has been recognised as a promising field in response to the accumulated pressure regarding environmental concerns, which also highlighted the importance of the lithium battery industry (Jing et al., 2021; Sun, 2021). Data show that the scale of China's lithium battery industry has exceeded 180 billion yuan in 2020, with promising growth potential (Qianzhan Industrial Research Institute, 2021). As a result, supporting the growth of this industry has attracted the attention of policymakers in China on a national scale.

The policy emphasis on the lithium battery industry in China has been reflected in the form of cultivating arrangements as well as investments, originating from the state, central and local governments. For example, in 2019, the *Lithium-ion Battery Industry Standard Conditions* (2018 Version) were issued, encouraging companies to strengthen top-level design and promote the upgrading of automation equipment. In 2020, the State Council issued the *New Energy Vehicle Industry Development Plan (2021–2035)*, pointing out that China will introduce battery technology breakthroughs to promote the development of the entire value chain, to build a high-efficiency power battery recycling system and to accelerate the promotion of power battery recycling legislation. Thanks to these measures, the growth of the lithium battery industry in China has been immensely promising, at least in terms of scale. According to the data (Qianzhan Industrial Research Institute, 2021), a total of 102 GWh lithium batteries were shipped in China in 2018, a yearly increase of 27%. In 2019, with an additional 29% increase, China's lithium-ion battery shipments was at 131.6 GWh. In 2020, this value reached 158.5 GWh. Thus, in terms of size, the lithium battery industry in China currently ranks first in the world.

However, in terms of performance in research and development (R&D) and technological advancements, the Chinese lithium battery industry still lags behind the best industries in the world, such as those in America and South Korea. The dependence on imports and lack of mastery of the core technology have become major obstacles to its development. This gap could be closed through stimulating R&D investments but is not a guaranteed solution. Existing studies have revealed that the effectiveness and efficiency of R&D investment, not just quantity, is the key to sustainable growth, especially for renewable energy industries (Ma et al., 2021; Mohsin et al., 2021; Zhou et al., 2022). In addition, the positive impact of efficient R&D investment on the sustainable growth of industries, especially in the introduction stage of their life cycle has been proven (Yoo et al., 2019). Another key characteristic of the lithium battery industry in China is that it is "top-heavy", thus making the performance of the leading enterprises crucial to the overall success of the industry. The industry has high barriers to enter due to various capabilities, such as technology, reputation and capital, thus giving evident advantages to the leading enterprises. With policymakers placing higher demands on products in terms of technological advancements, the market share will be further concentrated to the leading enterprises.

Therefore, to provide a clearer guidance on policymaking for the future of the industry, the current performance and potential problems in terms of R&D efficiency of the industry must be comprehensively understood. Considering the two key features of the industry, this study aims to address the following two research questions. Firstly, how efficient was the Chinese lithium battery industry in terms of R&D from 2010 to 2019? This question will be explored on the basis of the entire industry and individual enterprise. Secondly, how did the leading enterprises in the industry perform during the time, and how did their performance differ from the industry average?

To address these research questions, we consider data envelopment analysis (DEA) to be the most appropriate approach. The most common econometric methodologies used for efficiency and productivity related analysis are DEA and stochastic frontier analysis (SFA); both methods have proven to be helpful in efficiency-related studies (for example Liu et al. (2018) and Wang et al. (2020) for SFA, Niewerth et al. (2022) for DEA). However, we indicated that SFA can only be used when the production function model is known. Furthermore, it cannot accommodate multiple inputs and outputs, thus making it unsuitable for this research (Reinhard et al., 2000; Avkiran and Rowlands, 2008; Iglesias et al., 2010). In addition, adopting DEA provides three benefits for this study (Berg, 2010). Firstly, DEA is a nonparametric method, and a specific production function does not need to be set (Zhou et al., 2008; Wu et al., 2021). Given that the lithium battery industry is an emerging industry, its production function has not been thoroughly studied. Thus, nonparametric methods would be more suitable. Secondly, it is capable of handling multiple inputs and outputs, and the sources of inefficiency can be analysed and quantified for each evaluated unit (Wang and Huang, 2007; Han et al., 2017). This capability is particularly helpful, as we are also interested in the performance of individual firms, especially leading firms. Thirdly, DEA is proven to be useful in uncovering relationships that remain hidden (Tong and Ding, 2008; Fang et al., 2009). The reason for decision-making unit (DMU) inefficiency can be found by a projection analysis of each DMU; improvements can be planned for the future. As a result, DEA was selected for this study.

Consequently, we aim to make a three-fold contribution to knowledge in this study. The first contribution is that we provide overviews on the efficiency of R&D activities in China's lithium battery industry by demonstrating efficiency scores and returns to scale (RTS) from 2010 to 2019. This overview reveals changes and trends that may lead to problems in the industry's development. Our second contribution is that the individual performance of 22 listed lithium battery enterprises were analysed to determine the internal factors leading to the low-average technical efficiency. For the third contribution, five leading enterprises were selected to be compared with industry average, thus providing insights on whether they could still benefit from expanding their scale. Therefore, as this research focuses on finding the achievements and difficulties of Chinese listed lithium battery enterprises in R&D, we make suggestions on policymaking for the future R&D efficiency improvement of lithium battery enterprises.

The rest of the paper is organised as follows. We first review the relevant literature in Section 2, covering aspects of importance and measurement standards of R&D activities, application of DEA in R&D efficiency evaluation and existing studies on lithium battery. This portion is followed by a detailed description of the method applied and our sampling strategy in Section 3. The analysis, findings and discussions are then presented in Section 4. We conclude the paper by discussing the implications of the findings on policymaking.

### 2 Literature review

#### 2.1 Importance and measurement standards of R&D activities

R&D activities have proven crucial not only in enhancing the competitiveness of organisations but also in sustaining a healthy growth of industries. For emerging industries such as new energy, increasing R&D investment in terms of financial capital and personnel should be the policy action to consider (Lin and Xu, 2018). On this basis, when examining the Chinese lithium battery industry, the input of R&D (e.g., investments) and its outcomes (usually in the form of patents) are expected to be at a relatively high level. To obtain a better understanding of the R&D performance in this kind of industry, focusing solely on input or output level may be problematic. Hence, R&D efficiency could be a more suitable measure in the context, as it considers both inputs and outputs of R&D operations (Chiu et al., 2012).

The common methods used to study R&D efficiency include DEA, SFA and Malmquist index, to name a few. Among these approaches, DEA is considered a well-developed and beneficial method, especially in technology-intensive industries. By definition, DEA is a mathematical programming method that is applied to assess efficiency through multiple inputs and outputs (Yeh, 1996; Kozmetsky and Yue, 1998; Lin et al., 2018). The ground-breaking work done by Rousseau and Rousseau (1997) proved the potential of DEA-analysis in examining R&D activities.

#### 2.2 Application of DEA in R&D efficiency evaluation

Recent studies have also benefited from applying DEA and its variations with fruitful results. For instance, the SBM (slacks-based model)–DEA model has been adapted to evaluate the R&D investment efficiency of 16 South Korean local governments from 2010 to 2016 (Lee et al., 2020). Similarly, the DEA-Tobit model has been used to construct a benchmark for enterprise in the new energy vehicle industry in terms of their technological innovation efficiency from 2013 to 2018. The analysis was completed using a sample of 23 related Chinese companies (Fang et al., 2020).

Another stream of application of DEA is through multi-stage and network analysis. For example, a network DEA model incorporating both shared inputs and additional intermediate inputs has been constructed to evaluate the R&D efficiency and commercialisation efficiency of high-tech industries simultaneously in 29 provincial-level regions in China (Chen et al., 2020). Based on the two-stage efficiency values of different industries in the high-tech industry from 2014 to 2016, the two-stage DEA-Tobit model has been used to analyse empirically the five factors that affect the two-stage efficiency of the collaborative innovation of international industrial achievements (Zhang, 2020).

#### 2.3 Research on lithium battery industry

Existing studies have contributed to our understanding regarding related industries of lithium battery in different contexts. Research fields have mainly focused on key parts of manufacturing lithium battery, such as electrolyte (Shi et al., 2022; Bandyopadhyay et al., 2022) and anode materials (Lashari et al., 2022; Lv et al., 2022). Spent lithium batteries can cause pollution to the soil and seriously threaten the safety and property of people. Moreover, they contain valuable metals, such as cobalt and lithium. Thus, their recycling and treatment have important economic, strategic and environmental benefits (Shang et al., 2021). Methods for safely and effectively recycling lithium batteries to ensure they provide a boost to economic development have been widely investigated (Zhang et al., 2020; Zhu and Chen, 2020; Jing et al., 2021; Duan et al., 2022).

In conclusion, although the study on lithium battery has made promising achievements, the existing studies are mainly focusing on technical aspects with a lack of focus on the level of an industry. We must understand how efficient different enterprises are in managing their R&D to promote desirable outcomes in terms of technological advancements and generating commercial benefits. Approaching this aspect from a management perspective would also be helpful for policymaking in promoting the development of the industry. Additionally, the results from existing studies indicate the usefulness of DEA in studying the innovation efficiency of the overall industry as well as a performance benchmark for individual organisations. Therefore, we adopted DEA to evaluate the R&D efficiency of listed lithium battery enterprises in China. Then, five leading enterprises were selected and compared with the overall level to explore the differences in performance.

### 3 Research methodology

#### 3.1 Data envelopment analysis

To evaluate the R&D efficiency of Chinese lithium battery industry, this study adopts a standard DEA among listed enterprises. Specifically, we referred to two DEA models: The CCR (Charnes–Cooper–Rhodes) model

(Charnes et al., 1978) and the BCC (Banker–Charness–Cooper) model (Banker et al., 1984).

##### 3.1.1 CCR model

The CCR model is proposed under the assumption that production exhibits constant returns to scale (CRS) and obtains comprehensive technical efficiency (CRSTE). To judge a DMU's efficiency is to calculate whether it can fall on the production frontier of the production-possible set. We assume $n$ lithium battery enterprises, and they are regarded as DMUs to analyse the R&D efficiency. A DMU is expressed by $DMU_j$ ($j = 1, 2, ..., n$), and each $DMU_j$ contains $m$ inputs (R&D manpower and R&D expenses) $x_{ij}$ ($i = 1, 2, ..., m$, $x_{ij} > 0$) and $s$ outputs (technical improvements and economic benefits) $y_{rj}$ ($r = 1, 2, ..., s$, $y_{rj} > 0$). $u_r$ ($r = 1, 2, ..., s$) and $v_i$ ($i = 1, 2, ..., m$) are output and input weights, respectively. The input matrix, $\boldsymbol{X}_j = (x_{1j}, x_{2j}, ..., x_{mj})^{\mathrm{T}}$, and output matrix, $\boldsymbol{Y}_j = (y_{1j}, y_{2j}, ..., y_{sj})^{\mathrm{T}}$, represent the data of $DMU_j$.

The efficiency rate $h_j$ of a unit $DMU_j$ can be generally expressed as:

$$h_j = \frac{Weighted\ sum\ of\ outputs}{Weighted\ sum\ of\ inputs} = \frac{\sum_{r=1}^{s} u_r y_{rj}}{\sum_{i=1}^{m} v_i x_{ij}} \leqslant 1. \quad (1)$$

DEA analysis has two orientations, namely, input orientation or output orientation, depending on the nature of the problem. Considering the issues examined in this research, we have selected input orientation, which aims to minimise the combination of inputs to yield a combination of outputs. To solve the calculation difficulties and facilitate discussion, the relaxation variables $\boldsymbol{s}^-$ (input redundancy) and $\boldsymbol{s}^+$ (output insufficiency) and Archimedes infinitesimal $\varepsilon$ are introduced using linear programming and duality theory.

An input minimisation problem in the CCR model can be presented as:

$$\min\left[\theta - \varepsilon(\hat{\boldsymbol{e}}^{\mathrm{T}}\boldsymbol{s}^- + \boldsymbol{e}^{\mathrm{T}}\boldsymbol{s}^+)\right]$$
$$s.t.\begin{cases} \sum_{j=1}^{n} \boldsymbol{X}_j\lambda_j + \boldsymbol{s}^- = \theta\boldsymbol{X}_0 \\ \sum_{j=1}^{n} \boldsymbol{Y}_j\lambda_j - \boldsymbol{s}^+ = \boldsymbol{Y}_0 \\ \lambda_j \geqslant 0,\ j = 1, 2, ..., n \\ \boldsymbol{s}^+ \geqslant 0,\ \boldsymbol{s}^- \geqslant 0 \end{cases}, \quad (2)$$

where $\theta$ is the efficiency evaluation value and $\lambda$ is a vector parameter. $\lambda^0$, $\boldsymbol{s}^{-0}$, $\boldsymbol{s}^{+0}$, and $\theta^0$ are for the optimal solution of the above programming. The following conclusions can be obtained.

If $\theta^0 < 1$, then $DMU_{j0}$ is not effective, which indicates that the technical efficiency and scale efficiency of R&D activities are not optimal.

If $\theta^0 = 1$, but at least one of $\boldsymbol{s}^-$, $\boldsymbol{s}^+ \neq 0$, then $DMU_{j0}$ is weakly effective, and the optimal technical and scale efficiency is not achieved simultaneously. To achieve comprehensive efficiency, input can be reduced under the condition of constant output.

If $\theta^0 = 1$, and $\boldsymbol{s}^-$, $\boldsymbol{s}^+ = 0$, then $DMU_{j0}$ is effective, and the optimal technical efficiency and optimal scale efficiency are achieved simultaneously. The input resources are fully utilised, and the output is maximised.

##### 3.1.2 BCC model

The BCC model assumes the presence of variable returns to scale (VRS) (Wang and Huang, 2007) and obtains pure technical efficiency (VRSTE) and scale efficiency (scale), respectively. Compared with the CCR model, the BCC model adds $\sum_{j=1}^{n}|\lambda_j| = 1$ to the constraint condition (represents VRS). The conclusion of the BCC model is similar to that of the CCR model mentioned above. The relationship between CCR and BCC models is $CRSTE = VRSTE \times scale$.

#### 3.2 Inputs and outputs

We regard R&D investment as the input of our model; this input includes R&D expenses and R&D manpower. Furthermore, R&D expenses include R&D expenditure and the proportion of R&D expenditure (R&D expenditure/operating income). The R&D expenditure refers to the total R&D expense, covering all projects involving both internal and external ones supported by the firm. The R&D expenditure input index has been widely used and found to be suitable in previous studies (Zhong et al., 2011; Chun et al., 2015; Han et al., 2017). R&D manpower includes the number of technical personnel and the proportion of technical personnel (number of technical personnel/total number of employees). The R&D personnel input figure includes all staff engaged in either fundamental research, application research or experimental development (Zhong et al., 2011). The number of research staff on activities can be taken as the R&D manpower input index. In the absence of this data, the number of technical personnel is adopted to present the number of R&D personnel; this approach has also been adopted in previous studies (Hollanders and Celikel Esser, 2007).

We considered two aspects in the output of the model. The initial, direct outcomes of R&D investment are technical improvements. Here, patent data may be the most appropriate in capturing it (Wang and Huang, 2007; Guan and Chen, 2010). Although not all inventions are patentable or patented and the inventions patented have different qualities (Griliches, 1990), previous studies indicate that patents provide a fairly reliable measure of R&D activities (Pakes and Griliches, 1980; Acs et al., 2002). Therefore, this study employed the quantity of patent applications to measure technical improvements. In addition, the economic benefit is the key purpose of a company's R&D investment behaviour. Operating revenue and net profit can show the business value brought by the results of R&D activities after they are put into the market in the most intuitive form (Cao, 2020). Moreover, they can measure the profitability, growth and sustainability of China's listed lithium battery enterprises. More net profit indicates that the R&D and operation management benefits of the enterprise are good, which can reflect the actual profitability of the R&D and operation activities of the enterprise. Therefore, this study employed operating income and net profit as economic benefit indicators.

Given the time needed to complete an R&D project, introduce products to market (e.g., packaging, pricing and marketing) and gain a market share, a sector-dependent time lag occurs for the economic outcomes in evaluating R&D following the initial investment (Kafouros and Wang, 2008). According to the results of previous research on R&D efficiency, this period ranges from one to two years (Hollanders and Celikel Esser, 2007). Combined with the data collection situation, we decided to adopt a one-year R&D investment lag period, that is, the data on R&D investment were from 2010–2019, and the data of R&D output were from 2011–2020 to reflect this "lag period".

#### 3.3 Sampling and data collection

Existing research reports on related industries to lithium battery were consulted in the sampling process for this study. The factors considered in selecting samples include 1) market share, 2) listing years and 3) data integrity. A total of 22 listed lithium battery enterprises were eventually identified as the research objects. Data were collected according to Table 1, covering 2010 to 2019. The main data sources included government statistical databases, established commercial databases and corporate annual reports. The specific sources of data are shown in Table 1.

In addition to the initial sampling, we identified five enterprises as leading enterprises within the sample. At present, China's power lithium battery industry has a large number of listed enterprises, which are distributed in various industrial chains. We finally selected one listed enterprise of electric cells and battery packs (BYD), two listed enterprises of lithium raw materials (Tianqi Lithium and Ganfeng Lithium), one listed enterprise of anode materials (Hunan Zhongke Electric) and one listed enterprise of cathode materials (Beijing Easpring Material Technology) as the research samples of leading enterprises. The details of the five leading enterprises are shown in Table 2.

**Table 1** R&D efficiency evaluation index system for listed lithium battery enterprises

| Index category | Standard level | Index name | Data source |
|---|---|---|---|
| Input index | R&D manpower | The number of technical personnel | Corporate annual report |
| | | The proportion of technical personnel | Corporate annual report |
| | R&D expenses | R&D expenditure | Corporate annual report |
| | | The proportion of R&D expenditure | Corporate annual report |
| Output index | Technical improvement | The number of patent applications | State Intellectual Property Office |
| | Economic benefit | Operating income | Corporate annual report |
| | | Net profit | Corporate annual report |

**Table 2** Introduction of five leading enterprises

| Industrial chain link | Name | Main points |
|---|---|---|
| Cell and battery pack | BYD | Establish the world's leading technical and cost advantages in the field of power batteries |
| Lithium raw materials | Tianqi Lithium | It is one of the few enterprises in the world that simultaneously distribute two kinds of raw material resources: High-quality lithium mine and salt lake brine mine |
| | Ganfeng Lithium | It is the world's leading lithium ecological enterprise, with the production capacity of more than 40 kinds of lithium compounds and metal lithium products in five categories |
| Anode materials | Hunan Zhongke Electric | Graphite powder processing technology is internationally advanced; heat treatment process and graphite composite technology are leading in China |
| Cathode materials | Beijing Easpring Material Technology | Leading enterprise in lithium battery cathode material industry |

## 4 Data analysis and discussion

### 4.1 Overall efficiency of China's lithium battery industry

To understand the changes of R&D efficiency of the whole lithium battery industry, we first normalised the data of 22 listed lithium battery enterprises. Then, we aggregated the input and output indexes of the processed 22 enterprises to represent the input and output indexes of the whole lithium battery industry. Finally, we obtained industry data spanning 10 years for calculation. The R&D efficiency across the lithium battery industry was examined through data from 2010 to 2019, as presented in Table 3. An overall examination indicates that the R&D investment efficiency was mostly unchanged despite the rising R&D expenditure over the period.

The results obtained by applying DEA models were the relative efficiency values rather than the absolute efficiency values; the size of the values depended on the samples analysed together. The efficiency scores in Table 3 are the results obtained by analysing the input and output indexes of the lithium battery industry in the past 10 years. Therefore, they could not represent a direct indication in terms of the performance of the entire industry. Nevertheless, we could draw insights by comparing the efficiency score across the past 10 years to understand the changes. The only noticeable change was the decline in R&D efficiency in 2011, 2014, 2016 and 2018, which appeared to have resulted from reductions in scale efficiency (SE). However, three of the R&D efficiency reductions were related to increasing returns to scale (IRS), and one was related to diminishing returns to scale (DRS). Except for these four years, all the other R&D investment efficiencies from 2010 to 2019 were unchanged. The potential conclusion is that even with more than 10 years of development, the R&D investment efficiency in China's lithium battery industry has not exhibited any dramatic improvement.

**Table 3** Efficiency scores and RTS of the whole lithium battery industry in 2010–2019

| Year | Technical efficiency | Pure technical efficiency | Scale efficiency | RTS |
|---|---|---|---|---|
| 2010 | 1.000 | 1.000 | 1.000 | – |
| 2011 | 0.981 | 0.986 | 0.995 | irs |
| 2012 | 1.000 | 1.000 | 1.000 | – |
| 2013 | 1.000 | 1.000 | 1.000 | – |
| 2014 | 0.986 | 1.000 | 0.986 | irs |
| 2015 | 1.000 | 1.000 | 1.000 | – |
| 2016 | 0.997 | 1.000 | 0.997 | drs |
| 2017 | 1.000 | 1.000 | 1.000 | – |
| 2018 | 0.960 | 0.981 | 0.978 | irs |
| 2019 | 1.000 | 1.000 | 1.000 | – |
| Average | 0.992 | 0.997 | 0.996 | |

Notes: –: constant return to scale; irs: increasing return to scale; drs: decreasing return to scale.

### 4.2 Patents performance

Figure 1 presents an overview of the changes in data regarding R&D indicators collected in this study. The growth ratio was calculated to support the DEA results of R&D efficiency. Accordingly, the result suggested a disappointing prospect for the development of China's lithium battery industry investment. Although increasing R&D expenditure appeared to be correlated with a dramatic increase of operating income, knowledge output remains limited in terms of the increase in the number of patent applications. Moreover, the net profit has not increased significantly. In turn, this finding may suggest that although increasing R&D investment (inputs) appears to be related to the increase of operating income, it does not stem from the increase in the number of patent applications, which indicates that the level of innovation in the lithium battery industry has not improved.

### 4.3 Efficiency scores of 22 enterprises

#### 4.3.1 Technical efficiency (TE) and pure technical efficiency (PTE)

The results in Fig. 2 and Table 3 are obtained by using different samples and represent different meanings. Table 3 shows the results obtained by comparing the input and output indexes of the entire lithium battery industry in the past 10 years. Figure 2 was generated by comparing the input and output indexes of all 22 listed lithium battery enterprises individually in the past 10 years (220 DMUs in total) with calculations made on the

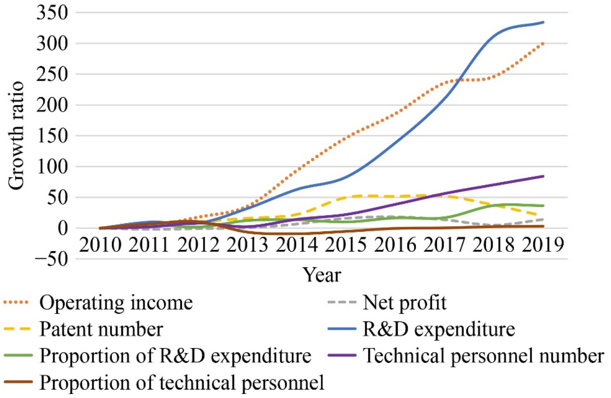

**Fig. 1** The growth ratio of R&D investment inputs and outputs (Note: The calculation of growth ratio is based on the data of 2010, all the indicators from the other years compared with the data from 2010. For example, the net profit growth ratio 2019 = (net profit 2019 − net profit 2010)/net profit 2010).

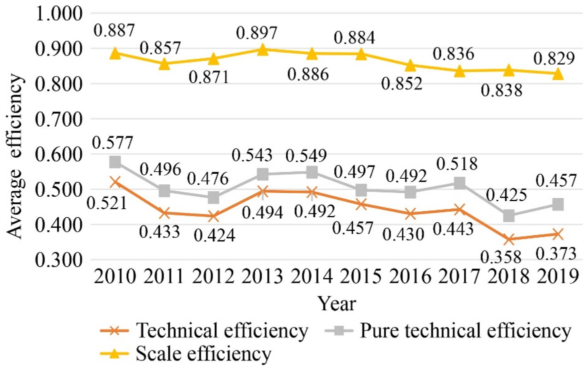

**Fig. 2** The changing trend of average efficiency scores of 22 listed lithium battery enterprises from 2010 to 2019.

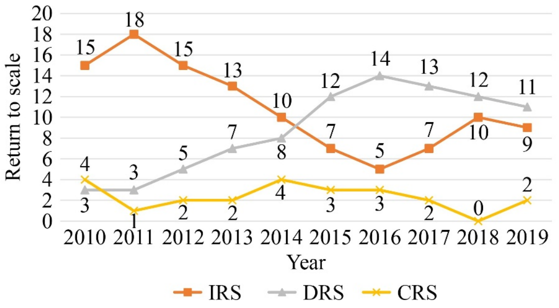

**Fig. 3** The changing trend of the RTS of 22 listed lithium battery enterprises from 2010 to 2019.

basis of the average efficiency score of 22 enterprises in each year. Thus, Fig. 2 demonstrates how the average efficiency score of the 22 enterprises changed over time. The average TE score of 22 listed lithium battery enterprises was only 0.442, thus indicating that the overall technical efficiency level is very low.

Specifically, the PTE scores reflect the pure R&D investment efficiency excluding scale effects. During the experimental period, the average PTE score of 22 listed lithium battery enterprises was 0.503, slightly higher than the average TE score. Furthermore, the fluctuations of the average PTE score were similar to the average TE score, and both were relatively low. This outcome implies that low technical efficiency may be affected by pure technical efficiency.

##### 4.3.2 Scale efficiency (SE)

Scale efficiency (SE) scores reflect various classes of returns to the scale of R&D investment. Accordingly, the average SE score of 22 listed lithium battery companies was 0.864, which was significantly higher than the average TE and the average PTE scores. In addition, the average SE score of 22 listed companies has changed relatively smoothly throughout the 10 years and has been at a relatively high level. This outcome indicates that scale efficiency is not the main reason for the low technical efficiency.

Figure 3 presents our result on scale efficiency indicators. Further analysis of the SE data indicates that RTS metrics could provide useful indices for the management of R&D investment efficiency. RTS have three possible classes: Decreasing (DRS), increasing (IRS) and constant (CRS). CRS is indicated by an SE score of 1; DRS is signified by a decrease in the relative output for a given incremental input and an associated decline in the consequent revenue/profit; and IRS is signified by an increase in the relative output for a given incremental input. Figure 3 shows that in 2019, compared with 2010, the number of companies suffering from DRS has greatly increased, accounting for half of the total number of companies; in addition, the number of companies with IRS has decreased, and the number of companies with CRS has been stable at a low level. These observations show that fewer and fewer companies rely solely on expanding scale to obtain additional economic benefits. In the future, lithium battery companies need to rely on continuous improvement of technological innovation capabilities to obtain additional economic benefits instead of blindly expanding production scale.

##### 4.3.3 The relationship between TE and PTE

A closer examination of the relationship between PTE and TE shows that the fluctuations and trend of the average PTE are similar to that of the average TE. The data in Fig. 2 present the average efficiency scores of 22 listed lithium battery enterprises every year. However, the figure does not show the specific efficiency scores of the 22 enterprises. To verify the relationship between the two further, we selected the 2010 and 2019 R&D efficiency scores of the 22 enterprises for analysis (Table 4).

In addition, Figs. 4(a) and 4(b) present the distribution of PTE and TE scores in plots, indicating their relationship to the average PTE and TE scores (solid lines).

According to Figs. 4(a) and 4(b), the enterprises in Zone A exhibit both high PTE and TE scores. Enterprises in Zone B show high PTE scores but low TE scores. Zone C enterprises exhibit low scores on both PTE and TE. Zone D has few enterprises, thus making the high TE score with a low PTE level an uncommon occurrence. The positive relationship between TE and PTE scores could be observed. The relationship is much stronger in Fig. 4(a), which suggests that the PTE level is more important in improving the TE score. It also highlights the importance of PTE improvement as a key management index for the increasing of the overall R&D investment efficiency level within China's listed lithium battery enterprises.

**Table 4** Efficiency scores of R&D investments in 22 China's listed lithium battery enterprises in 2010 and 2019, respectively

| Number | Name | TE | | PTE | | SE | | RTS | |
|---|---|---|---|---|---|---|---|---|---|
| | | 2010 | 2019 | 2010 | 2019 | 2010 | 2019 | 2010 | 2019 |
| 1 | Guangdong Fenghua Advanced Technology | 0.457 | 0.170 | 0.491 | 0.212 | 0.931 | 0.801 | irs | drs |
| 2 | Hengdian Group DMEGC Magnetics | 0.286 | 0.330 | 0.286 | 0.523 | 0.999 | 0.631 | – | drs |
| 3 | Guoxuan High-tech | 0.397 | 0.115 | 0.532 | 0.118 | 0.747 | 0.974 | irs | irs |
| 4 | Suzhou Good-Ark Electronics | 0.326 | 0.331 | 0.436 | 0.396 | 0.749 | 0.837 | irs | irs |
| 5 | Sinoma Science & Technology | 0.228 | 0.443 | 0.248 | 0.950 | 0.918 | 0.466 | irs | drs |
| 6 | Do-Fluoride Chemicals | 0.483 | 0.266 | 0.573 | 0.274 | 0.843 | 0.971 | irs | irs |
| 7 | Ganfeng Lithium | 0.632 | 0.829 | 0.782 | 0.895 | 0.809 | 0.926 | irs | drs |
| 8 | Tianqi Lithium | 0.589 | 0.277 | 0.830 | 0.503 | 0.710 | 0.551 | irs | irs |
| 9 | BYD | 0.644 | 1.000 | 0.685 | 1.000 | 0.941 | 1.000 | irs | – |
| 10 | Eve Energy | 0.520 | 0.250 | 0.665 | 0.509 | 0.782 | 0.492 | irs | drs |
| 11 | Hunan Zhongke Electric | 0.781 | 0.315 | 0.793 | 0.375 | 0.985 | 0.840 | irs | irs |
| 12 | Beijing Easpring Material Technology | 0.675 | 0.433 | 0.782 | 0.487 | 0.863 | 0.888 | irs | irs |
| 13 | Sunwoda Electronic | 0.237 | 0.326 | 0.277 | 0.379 | 0.856 | 0.860 | irs | drs |
| 14 | Wanxiang Qianchao | 0.210 | 0.336 | 0.268 | 0.337 | 0.783 | 0.994 | drs | irs |
| 15 | Shenzhen CLOU Electronics | 0.435 | 0.123 | 0.441 | 0.126 | 0.988 | 0.975 | irs | irs |
| 16 | Shenzhen Topband | 0.511 | 0.146 | 0.515 | 0.190 | 0.991 | 0.771 | irs | drs |
| 17 | Zhejiang Unifull Industrial Fibre | 1.000 | 0.304 | 1.000 | 0.420 | 1.000 | 0.723 | – | irs |
| 18 | Zhejiang Narada Power Source | 0.596 | 0.398 | 0.624 | 0.400 | 0.955 | 0.996 | irs | drs |
| 19 | China CSSC Holdings | 1.000 | 0.461 | 1.000 | 0.585 | 1.000 | 0.788 | – | drs |
| 20 | Jiangsu Zhongtian Technology | 0.408 | 1.000 | 0.412 | 1.000 | 0.990 | 1.000 | drs | – |
| 21 | Neusoft Corporation | 0.042 | 0.047 | 0.063 | 0.059 | 0.665 | 0.786 | drs | drs |
| 22 | Shenzhen Capchem Technology | 1.000 | 0.304 | 1.000 | 0.316 | 1.000 | 0.960 | – | drs |
| Mean | | 0.521 | 0.373 | 0.577 | 0.457 | 0.887 | 0.829 | | |

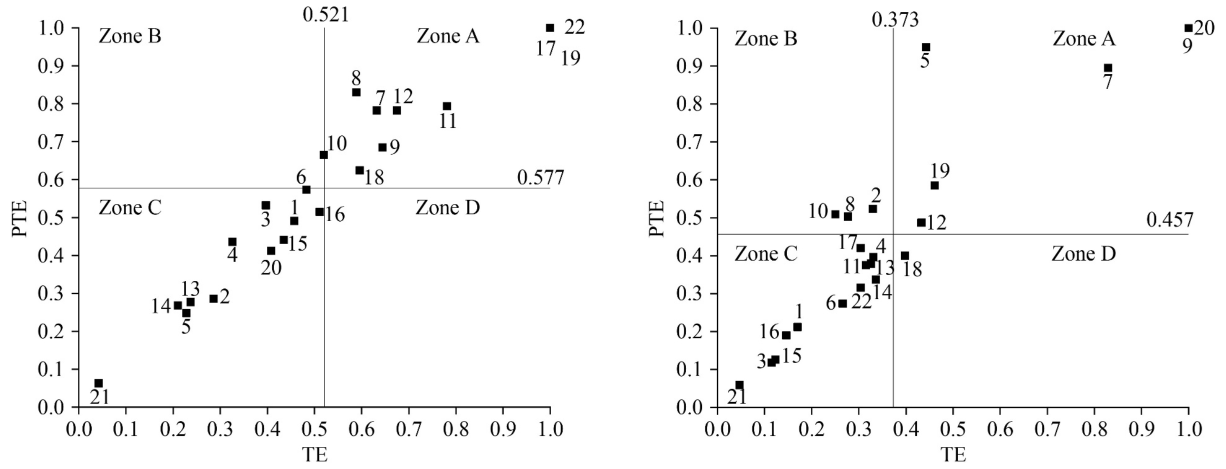

(a) The comparison of PTE and TE scores of 22 enterprises in 2010 (b) The comparison of PTE and TE scores of 22 enterprises in 2019

**Fig. 4** The comparison of PTE and TE scores of 22 enterprises in 2010 and 2019, respectively.

#### 4.4 Efficiency scores of leading enterprises

##### 4.4.1 Technical efficiency (TE) and pure technical efficiency (PTE)

Table 5 showcases the performance of the five leading enterprises identified. During the observed period, the average TE score of the five companies was 0.659, which was higher than that of the 22 enterprises of 0.442. These scores indicated that the R&D investment efficiency of the leading enterprises was higher than the industrial average. Table 6 shows that during the experimental period, the average PTE score of the five companies was 0.724, higher than that of the 22 enterprises of 0.503, which indicated that the technological innovation level of leading enterprises was relatively high. According to the average TE and PTE scores from 2010 to 2019, only BYD and Tianqi Lithium exchanged rankings, while the rankings of the other three companies remained unchanged. The specific rankings and scores can be seen in Tables 5 and 6.

##### 4.4.2 Scale efficiency (SE)

Table 7 shows that during the experimental period, the average SE score of the five leading enterprises was 0.903, slightly higher than the average PTE score. Thus, the scores indicated that the scale efficiency of the leading enterprises was higher than the industry average.

Table 7 also demonstrates that the leading enterprises were in the situation of IRS most of the time. For example, in 2019, among the five leading companies, only BYD reached CRS, which means that it reached the best scale efficiency. Ganfeng Lithium were in the state with DRS, and the other three enterprises were in the state with IRS, which means that for leading companies, the efficiency of R&D investment could still be increased by expanding the scale of the company.

**Table 5** Technical efficiency scores of five leading enterprises from 2010 to 2019

| Year | BYD | Tianqi Lithium | Hunan Zhongke Electric | Ganfeng Lithium | Beijing Easpring Material Technology |
|---|---|---|---|---|---|
| 2010 | 0.644 | 0.589 | 0.781 | 0.632 | 0.675 |
| 2011 | 0.578 | 0.567 | 0.671 | 0.472 | 0.534 |
| 2012 | 0.624 | 0.505 | 0.692 | 0.418 | 0.624 |
| 2013 | 0.618 | 0.736 | 0.674 | 0.696 | 0.618 |
| 2014 | 0.736 | 0.783 | 0.734 | 0.706 | 0.679 |
| 2015 | 1.000 | 0.900 | 0.996 | 0.392 | 0.598 |
| 2016 | 1.000 | 1.000 | 1.000 | 0.470 | 0.470 |
| 2017 | 0.993 | 0.865 | 0.491 | 1.000 | 0.452 |
| 2018 | 0.922 | 0.507 | 0.363 | 0.275 | 0.391 |
| 2019 | 1.000 | 0.277 | 0.315 | 0.829 | 0.433 |
| Average | 0.812 | 0.673 | 0.672 | 0.589 | 0.547 |
| Rank | 1 | 2 | 3 | 4 | 5 |

**Table 6** Pure technical efficiency scores of five leading enterprises from 2010 to 2019

| Year | BYD | Tianqi Lithium | Hunan Zhongke Electric | Ganfeng Lithium | Beijing Easpring Material Technology |
|---|---|---|---|---|---|
| 2010 | 0.685 | 0.830 | 0.793 | 0.782 | 0.782 |
| 2011 | 0.642 | 0.793 | 0.690 | 0.602 | 0.611 |
| 2012 | 0.629 | 0.719 | 0.721 | 0.529 | 0.663 |
| 2013 | 0.628 | 0.946 | 0.715 | 0.702 | 0.675 |
| 2014 | 0.736 | 1.000 | 0.748 | 0.706 | 0.717 |
| 2015 | 1.000 | 0.979 | 1.000 | 0.399 | 0.652 |
| 2016 | 1.000 | 1.000 | 1.000 | 0.626 | 0.505 |
| 2017 | 1.000 | 1.000 | 0.521 | 1.000 | 0.493 |
| 2018 | 0.924 | 0.612 | 0.409 | 0.285 | 0.506 |
| 2019 | 1.000 | 0.503 | 0.375 | 0.895 | 0.487 |
| Average | 0.824 | 0.838 | 0.697 | 0.653 | 0.609 |
| Rank | 2 | 1 | 3 | 4 | 5 |

**Table 7** Scale efficiency scores of five leading enterprises from 2010 to 2019

| Year | BYD | | Tianqi Lithium | | Hunan Zhongke Electric | | Ganfeng Lithium | | Beijing Easpring Material Technology | |
|---|---|---|---|---|---|---|---|---|---|---|
| 2010 | 0.941 | irs | 0.710 | irs | 0.985 | irs | 0.809 | irs | 0.863 | irs |
| 2011 | 0.899 | irs | 0.714 | irs | 0.972 | irs | 0.784 | irs | 0.874 | irs |
| 2012 | 0.992 | irs | 0.703 | irs | 0.960 | irs | 0.790 | irs | 0.941 | irs |
| 2013 | 0.984 | drs | 0.777 | irs | 0.942 | irs | 0.991 | irs | 0.916 | irs |
| 2014 | 1.000 | – | 0.783 | irs | 0.981 | irs | 1.000 | – | 0.948 | irs |
| 2015 | 1.000 | – | 0.919 | irs | 0.996 | irs | 0.984 | drs | 0.917 | irs |
| 2016 | 1.000 | – | 1.000 | – | 1.000 | – | 0.751 | drs | 0.931 | irs |
| 2017 | 0.993 | drs | 0.865 | drs | 0.943 | irs | 1.000 | – | 0.918 | irs |
| 2018 | 0.998 | irs | 0.828 | irs | 0.889 | irs | 0.963 | drs | 0.773 | irs |
| 2019 | 1.000 | – | 0.551 | irs | 0.840 | irs | 0.926 | drs | 0.888 | irs |
| Average | 0.981 | | 0.785 | | 0.951 | | 0.900 | | 0.897 | |
| Rank | 1 | | 5 | | 2 | | 3 | | 4 | |

### 4.5 Discussion

Our findings provided insights on R&D efficiency performance for the lithium battery industry in China as well as for individual firms. Although some results do not show any potential contributions for the industry, three key points that are relevant to further policymaking are worth noting.

#### 4.5.1 R&D investment did not bring significant improvement in technical productivity

The first and most striking result of the analysis is the overall efficiency performance of the industry. Our results indicated that the R&D investment efficiency in China's lithium battery industry was nearly unchanged from 2010 to 2019 (see Table 3). Unsurprisingly, the overall R&D investment performance of the lithium battery industry did not show any increase even though the R&D expenditure steadily increased during the examination period. Further analysis revealed that the increased R&D expenditure was associated with a dramatic increase (see Fig. 1) in operating income; yet, the number of patent applications and the net profit had a small increase. This finding suggests that the increase of R&D inputs has brought an evident improvement to the operating incomes, but it has not yet led to improvements in the areas of technological advancement and production efficiency. This result is consistent with the current state of the industry, where it ranks first in terms of scale but lags on innovativeness.

#### 4.5.2 Low pure technical efficiency is the main factor restricting the overall R&D efficiency

We measured the technical efficiency, pure technical efficiency and scale efficiency of 22 listed lithium battery enterprises. The average TE score of 22 enterprises during the period was only 0.442, thus indicating that the overall technical efficiency level was low. The average PTE score was 0.503, and the average SE score was 0.864. Among the 22 enterprises, the number of enterprises suffering DRS has increased over the past 10 years. The relationship between PTE and TE was also analysed, and the results showed an evident positive correlation between PTE and TE. Therefore, we conclude that low pure technical efficiency is the main factor restricting the improvement on the overall R&D efficiency. This observation indicates that further support may be needed to help enterprises not only increase the level of R&D investment but also focus on improving the R&D operations and process to achieve a higher level of efficiency. Blindly expanding in scale could be a dangerous action in promoting the growth of the industry.

#### 4.5.3 Leading enterprises can still improve R&D efficiency by expanding their scale

The lithium battery industry is a technology-intensive industry, and the leading enterprises have demonstrated an obvious scale and technical advantages. The analysis on the R&D efficiency of the leading enterprises shows that the average TE score of leading enterprises during the experimental period was 0.659, the average PTE score was 0.724, and the average SE score was 0.903. These values are all higher than the average scores of the 22 listed lithium battery enterprises, thus indicating a better R&D efficiency performance of the leading enterprises. This outcome is also consistent with the understanding that the structure of this industry is "top heavy". Thus, the practice of the leading enterprises is worth exploring, and their experiences should be used as best practices in helping other firms in the industry increase their R&D efficiency.

In addition, an analysis of the RTS of leading enterprises shows that the five leading enterprises are experiencing IRS most of the time. Therefore, different from other enterprises in the industry, leading enterprises can still obtain benefits by expanding their scale, which may also contribute to early capital accumulation and policy support.

### 5 Conclusions and policy implications

#### 5.1 Conclusions

This study aims to understand the efficiency of Chinese enterprises working on lithium battery in terms of R&D during 2010 to 2019. We are also interested in how leading enterprises have performed compared with the industrial average during this period. Building our model on the basis of the classic CCR and BCC model of DEA, our results indicate a need for improvement for enterprises in the lithium battery industry of China. We conclude that most of the enterprises in our sample are suffering from DRS. In contrast, the performance of leading enterprises is superior, and they can still obtain benefits by expanding their scale. Our findings have contributed to making suggestions for policy as well as future research.

#### 5.2 Policy implications

Our suggestion for policymakers is that supporting technologically intensive sectors should entail more than simply increasing investment scale. Rather, it should also encompass assisting businesses in developing efficient managerial processes for R&D. Moreover, leading enterprises should be regarded as best practices that can help other enterprises to improve their R&D efficiency.

Getting to know industry efficiency is crucial for designing tailored energy efficiency and adaptation policies for policymakers and managers working on the healthy and sustainable development of China's lithium battery industry. As we only viewed R&D in general, our suggestions could also be linked with suggestions from other studies that focus on specific aspects of sustainable development, such as green supply chain (Liu et al., 2022) and manufacturing process (Yang et al., 2022), to form a comprehensive policy plan.

Our findings are already consistent with the current policy actions in China. In Column 1 of the *New Energy Vehicle Industry Development Plan (2021–2035)* released by the State Council, the battery technology breakthrough action was mentioned. The Plan also proposed to support the development of ecological leading enterprises, that is, giving play to the leading enterprises, cultivating several upstream and downstream collaborative innovations and financing all sizes of enterprises. These policies are in line with our suggestion. However, actions specific to different types of enterprises could be better evidenced. In addition to the central policies, the provinces have also promulgated policies on the development of lithium batteries in recent years. Shanghai, Zhejiang, Tianjin and other provinces and cities have put forward relevant goals for breakthroughs in power battery materials and technologies. These goals have become an important factor in promoting the growth of the industry that should be recognised. For example, similar to the suggestion of this study, Fujian Province has proposed to support leading power battery enterprises to expand the production and marketing scale and continue to maintain product technology leadership.

#### 5.3 Limitations and future research

Despite the valuable insights obtained by the study, our research has several limitations. In this study, we adapted the traditional self-evaluation DEA method to evaluate the R&D efficiency of lithium battery enterprises. However, this method may exaggerate the effects of several inputs or outputs of the evaluated DMU, thus resulting in unrealistic results (Wu et al., 2021). For future studies, better efficiency evaluation methods, such as cross-efficiency evaluation (CREE), could be explored. Additional fruitful insights may be generated by taking advantage of the recent development of using big data and building new evaluation methods (for example, Yang and Wang, 2020).

Moreover, this research only examined the R&D efficiency performance of Chinese lithium battery enterprises, without comparative analysis with technology-leading countries. Future studies could consider comparing the lithium battery industries in different regions, together with its supporting policies, to obtain more insights on the performance and impact of different policies.

**Open Access** This article is licensed under a Creative Commons Attribution 4.0 International License, which permits use, sharing, adaptation, distribution and reproduction in any medium or format, as long as you give appropriate credit to the original author(s) and the source, provide a link to the Creative Commons licence, and indicate if changes were made.

The images or other third party material in this article are included in the article's Creative Commons licence, unless indicated otherwise in a credit line to the material. If material is not included in the article's Creative Commons licence and your intended use is not permitted by statutory regulation or exceeds the permitted use, you will need to obtain permission directly from the copyright holder. To view a copy of this licence, visit http://creativecommons.org/licenses/by/4.0/.
